# Optimism is not associated with two indicators of DNA methylation aging

**DOI:** 10.18632/aging.102090

**Published:** 2019-07-18

**Authors:** Eric S. Kim, Kelvin Fong, Lewina Lee, Avron Spiro, Joel Schwartz, Eric Whitsel, Steve Horvath, Cuicui Wang, Lifang Hou, Andrea A. Baccarelli, Yun Li, James Stewart, JoAnn E. Manson, Francine Grodstein, Dawn L. DeMeo, Laura D. Kubzansky

**Affiliations:** 1 Department of Social and Behavioral Sciences, Harvard T.H. Chan School of Public Health, Boston, MA 02115, USA; 2 Lee Kum Sheung Center for Health and Happiness, Harvard T.H. Chan School of Public Health, Boston, MA 02115, USA; 3 Program on Integrative Knowledge and Human Flourishing, Institute for Quantitative Social Science, Harvard University, Cambridge, MA 02138, USA; 4 School of Forestry and Environmental Studies, Yale University, New Haven, CT 06511, USA; 5 VA Boston Healthcare System, Boston, MA 02130, USA; 6 Department Psychiatry, Boston University School of Public Health, Boston, MA 02118, USA; 7 Massachusetts Veterans Epidemiology Research and Information Center (MAVERIC), VA Boston Healthcare System, Boston, MA 02130, USA; 8 Department of Epidemiology, Boston University Schools of Public Health and Medicine, Boston, MA 02118, USA; 9 Department of Environmental Health, Harvard T.H. Chan School of Public Health, Boston, MA 02115, USA; 10 Department of Epidemiology, Harvard T.H. Chan School of Public Health, Boston, MA 02115, USA; 11 Department of Epidemiology, Gillings School of Global Public Health, Chapel Hill, NC 27599, USA; 12 Department of Medicine, School of Medicine, University of North Carolina, Chapel Hill, NC 27599, USA; 13 Department of Human Genetics, University of California, Los Angeles, CA 90095, USA; 14 Department of Biostatistics, David Geffen School of Medicine, University of California, Los Angeles, CA 90095, USA; 15 Department of Preventive Medicine, Northwestern Feinberg School of Medicine, Northwestern University, Chicago, IL 60611, USA; 16 Department of Environmental Health Sciences, Columbia Mailman School of Public Health, New York, NY 10032, USA; 17 Department of Genetics, University of North Carolina, Chapel Hill, NC 27599, USA; 18 Department of Biostatistics, University of North Carolina, Chapel Hill, NC 27599, USA; 19 Department of Computer Science University of North Carolina, Chapel Hill, NC 27599, USA; 20 Cardiovascular Program, Department of Epidemiology, University of North Carolina Gillings School of Global Public Health, Chapel Hill, NC 27599, USA; 21 Department of Medicine, Brigham and Women’s Hospital, Harvard Medical School, Boston, MA 02115, USA; 22 Channing Division of Network Medicine, Brigham and Women’s Hospital, Boston, MA 02115, USA

**Keywords:** psychological well-being, optimism, health psychology, epigenetics, healthy aging, DNA methylation

## Abstract

Evidence indicates associations between higher optimism and reduced risk of age-related conditions and premature mortality. This suggests optimism is a positive health asset, but research identifying potential biological mechanisms underlying these associations remains limited. One potential pathway is slower cellular aging, which may delay age-related deterioration in health. Data were from the Women’s Health Initiative (WHI) (N=3,298) and the Veterans Affairs Normative Aging Study (NAS) (N=514), and included dispositional and explanatory style optimism measures. We evaluated whether higher optimism was associated with metrics suggestive of less cellular aging, as indicated by two DNA methylation algorithms, intrinsic (IEAA) and extrinsic epigenetic age acceleration (EEAA); these algorithms represent accelerated biologic aging that exceeds chronological age. We used linear regression models to test our hypothesis while considering several covariates (sociodemographics, depressive symptoms, health behaviors). In both cohorts, we found consistently null associations of all measures of optimism with both measures of DNA methylation aging, regardless of covariates considered. For example, in fully-adjusted models, dispositional optimism was not associated with either IEAA (WHI:β=0.02; 95% Confidence Interval [CI]:-0.15-0.20; NAS:β=-0.06; 95% CI:-0.56-0.44) or EEAA (WHI:β=-0.04; 95% CI: -0.26-0.17; NAS:β=-0.17; 95% CI: -0.80-0.46). Higher optimism was not associated with reduced cellular aging as measured in this study.

## Introduction

Aging and age-related health conditions have become critical public health issues. According to recent projections, the percentage of people aged >65 years in the United States is expected to increase by more than 60% over the next 15 years [1]. Growing evidence suggests that higher levels of optimism are associated with reduced risk of a wide range of age-related conditions such as cardiovascular events, lung function decline, cognitive impairment, and premature mortality (including both overall mortality and deaths due to heart disease, stroke, or cancer) [2–11]. For example, in a prospective study of 70,021 older women followed over 8 years, women in the highest (versus lowest quartile of optimism had a 38% reduced risk (95% confidence interval [CI]: 0.50, 0.76) of heart disease mortality and a 39% reduced risk (95% CI: 0.43, 0.85) of stroke mortality after adjusting for sociodemographic factors [2]. Prospective studies in other cohorts have reported similar findings [5,12–14].

Optimism, which has been defined either as the generalized expectation that good things will happen or according to the ways in which people explain the causes of good and bad events, may therefore be a powerful, positive health asset. Biologic mechanisms underlying the optimism-health associations are not yet well-established, but understanding biologic pathways could point to novel means of improving health in aging. Given the broad associations between optimism and health across disease endpoints, it is possible that optimism is related to systemic processes that affect multiple outcomes. Given consistent findings with age-related diseases, one candidate process is slower cellular aging, whereby optimism could reduce or delay age-related deterioration in health.

Recent work has identified DNA methylation (DNAm) as a strong component of biologic aging, and developed “epigenetic clocks” designed to capture methylation-based markers of aging. Horvath et al. proposed an “epigenetic clock” derived from age-associated methylation changes in 353 Cytosine-phosphate-Guanosine sites (CpGs) across the genome that are involved in important biological processes (e.g., DNA replication and repair, lipid metabolism, oxidative stress, and other chronic disease related processes) [15,16]. This clock score is well-validated across multiple cell types and tissues, and in epidemiologic studies it predicts cognitive function, lung function, grip strength, and premature mortality, among other outcomes [16–18]. Hannum et al. also derived an epigenetic clock score in a slightly different way, leveraging DNA methylation in blood at 71 CpG sites [15]. A measure of methylation age acceleration can be derived using either of these scores to capture a positive difference between DNA methylation and chronologic age.

Prior work has suggested epigenetic aging might explain observed relationships between negative psychological factors and health. For example, stress-related epigenetic aging has been proposed as a possible explanatory factor linking psychological stress and higher risk of developing age-related diseases, with several studies demonstrating higher levels of lifetime stress are associated with accelerated DNA methylation age (DNAm) [19,20]. While less work has examined positive psychological factors in relation to epigenetic aging, to assess potential protective effects on aging processes, given prior findings of protective effects in relation to chronic disease outcomes, such relations are plausible. Optimism may be associated with a slower rate of epigenetic aging if it either reduces stress exposure or buffers its effects – although it is important to note that optimism does not simply reflect the absence of stress and in fact may have independent effects on biological processes [6]. Optimism has been characterized as an asset or resource that people can utilize throughout life and across multiple domains [11,21,22]. Generally, psychological health assets like optimism, tend to be stable across time, although they can be responsive to life changes such as unemployment or divorce [23], or to interventions, such as activities that promote psychological well-being [24–26].

In the current study, we sought to test the hypothesis that higher optimism would be associated with metrics suggestive of less cellular aging, as indicated by two DNA methylation age algorithms, intrinsic (IEAA) and extrinsic epigenetic age acceleration (EEAA); these algorithms represent accelerated biologic aging that exceeds chronological age. Intrinsic epigenetic age acceleration captures properties of aging that are independent of cell type and organ whereas extrinsic epigenetic acceleration likely reflects both epigenetic variation and age-related changes in cell distributions in blood [27]. We used data from two ongoing epidemiologic cohorts that include women or men, the Women’s Health Initiative (WHI) and the VA Normative Aging Study (NAS). Based on prior work we identified relevant covariates for consideration including sociodemographic factors, health status, and health behaviors, which might confound or lie on the pathway between optimism and DNA methylation, as well as depression, which is correlated with both optimism and DNA methylation profiles [28].

## RESULTS

### Sample description

The WHI sample consisted of 3,298 women including 1,665 Whites, 961 Blacks, and 561 Hispanics, and 111 in the “Other” race/ethnicity category. Chronological age in the WHI sample ranged from 50-79 years (mean=64). Most women were married (or in marriage-like relationships: 56%), and most had education after high school (some college or an associate degree: 26%, or a college or graduate degree: 30%). The NAS sample consisted of 514 men (99% White) ranging in chronological age from 56-91 years (mean=73). Most men were married (76%), and had either a high school degree (20%) or college or graduate degrees (35%). Table 1, Table S1, and Table S2 [see Appendix A 1 for Table S1 and Table S2] provide additional details about participants.

**Table 1:** Characteristics of study participants at baseline – Women’s Health Initiative and Normative Aging Study.

	**Women’s Health Initiative (WHI)** (n=3,298)		**Normative Aging Study (NAS)** (n=514)	
	Optimism Levels		Optimism Levels	
**Characteristics**	Quartile 1 (n = 1,004)	Quartile 4 (n = 753)	1^st^ Quartile (n=129)	4^th^ Quartile (n=129)
**Demographic Factors**				
Mean Age (SD)	63.4 (7.2)	63.2 (7.2)	72.9 (6.6)	73.2 (6.4)
Race/Ethnicity (%)				
White	43.7	51.9	99.2	99.2
Black / African-American	28.1	33.2	0.8	0.8
Hispanic / Latino	23.7	12.4	0	0
Other	4.5	2.5	0	0
Missing	0	0	0	0
Marital Status (%)				
Marriage or marriage-like relationship	50.6	57.2	73.6	74.4
Divorced or single	25.0	23.5	16.3	15.5
Widowed	23.4	19.0	9.3	10.1
Missing	1.0	0.3	0.8	0
Education (%)				
Less than high school	32.4	14.6	5.4	2.3
High school graduate	22.2	13.9	20.9	20.9
Some college or associate degree	24.0	28.7	24.8	16.3
College or more	20.5	41.8	27.9	39.5
Missing	0.9	0.9	20.9	20.9
Income (%)				
WHI				
Less than $20,000	35.2	17.4		
$20,000 to $49,999	41.7	44.5		
$50,000 to $74,999	9.1	16.2		
$75,000 or more	6.6	15.7		
Missing	7.5	6.2		
NAS				
Less than $60,000			29.5	26.4
$60,000 to $69,999			19.4	19.4
$70,000 to $89,999			21.7	25.6
$90,000 or more			25.6	27.9
Don’t know			0	0
Missing			3.9	0.8
**Health Factors**				
Depressed (%)*				
Not depressed	73.5	93.4	68.2	96.1
Depressed	20.3	4.0	28.7	0.8
Missing	6.2	2.7	3.1	3.1
Chronic Condition (%)**				
No chronic condition	36.7	39.8	48.1	64.3
Chronic condition	52.1	49.4	51.9	35.7
Missing	11.3	10.8	0.0	0.0
**Health Behaviors**				
Smoking (%)				
Never smoker	52.5	52.6	28.7	33.3
Past smoker	34.7	37.3	69.0	62.8
Current smoker	12.1	8.6	2.3	3.9
Missing	0.8	1.5	0	0
Physical activity level (METS/week; %)				
<3.0	41.6	32.3	38.0	19.4
3.0-8.99	23.5	22.7	27.1	29.5
9.0-17.99	14.4	19.5	20.2	19.4
18.0-26.99	7.1	9.4	4.7	10.1
≥27	6.4	9.7	10.1	21.7
Missing	7.0	6.4	0	0
Diet				
WHI				
Mean Healthy Eating Index (SD)	63.6 (11.5)	65.7 (11.2)		
NAS				
Mean Fruit Intake (SD)			2.6 (1.8)	2.5 (1.6)
Mean Vegetable Intake (SD)			3.1 (1.9)	3.5 (2)
Current drinker (%)				
Non drinker	42.2	36.3	29.5	19.4
Current drinker	57.1	63.4	69.8	79.8
Missing	0.7	0.4	0.8	0.8
Body Mass Index (%)				
Normal (<24.9)	19.6	24.0	16.3	21.7
Overweight (25.0-29.9)	35.6	32.3	50.4	56.6
Obese (≥30.0)	44.3	42.6	33.3	21.7
Missing	0.5	1.1	0	0

**Notes -** *Depressive symptoms in WHI were measured using the Burnam Screening Algorithm, a questionnaire that includes 6 items from the Center for Epidemiologic Studies Depression Scale (CES-D) and two from the Diagnostic Interview Scale (DIS), with a cutoff of ≥0.06 indicating depression. Depressive symptoms in NAS were measured using the Brief Symptom Inventory (BSI), with a cutoff of ≥0.638 indicating depression

**Chronic conditions in WHI included: 1) hypertension, 2) high cholesterol, 3) cardiovascular disease, 4) diabetes, 5) stroke, 6) cancer. Chronic conditions in NAS included: 1) cardiovascular disease, 2) diabetes, 3) stroke, 4) cancer

### Optimism and DNA methylation age

In WHI, when considering both dispositional optimism and DNAm age as continuous variables, we observed no association of optimism with IEAA (mean difference, β=0.07; 95% CI: -0.10,0.24; Table 2); however, we did observe an association with EEAA (mean difference, β=-0.35; 95% CI: -0.55,-0.13) in unadjusted models such that higher optimism was associated with lower DNAm age. In models that adjusted for basic confounders, we observed no relation of optimism to IEAA (β=0.02; 95% CI: -0.16,0.19; Table 2) and associations with EEAA were attenuated and no longer statistically significant (β=-0.06; 95% CI: -0.28,0.16). After further adjusting for the full set of covariates, associations were not meaningfully different (Table 2).

**Table 2:** Mean differences (Regression Coefficients) for association between optimism (LOT-R) and DNA methylation age in WHI (n=3,298)*.

	Optimism				
Outcome	Continuous Optimism Score**	Quartile 1 (n = 1,004)	Quartile 2 (n = 809)	Quartile 3 (n = 732)	Quartile 4 (n = 753)
**Intrinsic Epigenetic Age Acceleration**	Mean Difference ß (95% CI)		Mean Difference ß (95% CI)	Mean Difference ß (95% CI)	Mean Difference ß (95% CI)
Unadjusted Model	0.07 (-0.10, 0.24)	Ref.	0.01 (-0.44, 0.46)	-0.01 (-0.48, 0.45)	0.13 (-0.33, 0.58)
Basic Confounders Model***	0.02 (-0.16, 0.19)	Ref.	-0.11 (-0.56, 0.35)	-0.16 (-0.64, 0.31)	-0.03 (-0.50, 0.45)
All Covariates Model****	0.02 (-0.15, 0.20)	Ref.	-0.06 (-0.52, 0.40)	-0.14 (-0.61, 0.34)	-0.01 (-0.49, 0.47)
**Extrinsic Epigenetic Age Acceleration**					
Unadjusted Model	-0.35 (-0.55, -0.13)	Ref.	-0.52 (-1.09, 0.06)	-0.93 (-1.52, -0.34)	-0.73 (-1.32, -0.15)
Basic Confounders Model***	-0.06 (-0.28, 0.16)	Ref.	-0.19 (-0.76, 0.38)	-0.53 (-1.13, 0.06)	-0.05 (-0.65, 0.54)
All Covariates Model****	-0.04 (-0.26, 0.17)	Ref.	-0.10 (-0.67, 0.47)	-0.43 (-1.02, 0.16)	-0.02 (-0.62, 0.58)

**Notes -** *All models adjusted for WHI substudy (EMPC or BAA23)

**Per 1 SD increase in LOT-R score

***Confounders model adjusts for: race, education, income, marital status, chronic conditions, depression,

****All covariates model additionally adjusts for: physical activity, smoking, BMI, diet, alcohol consumption, which may be intermediates or confounders

When evaluating the association between dispositional optimism and DNAm age in NAS, we observed a pattern of null findings in unadjusted as well in all the other models. For example, in our basic adjusted models, optimism was not associated with IEAA (β=-0.06; 95% CI: -0.55,0.42; Table 3) or EEAA (β=-0.23; 95% CI: -0.83,0.38).

**Table 3:** Mean differences (Regression Coefficients) for association between optimism (LOT) and DNA methylation age in NAS (n=514).

	Optimism				
Outcome	Continuous Optimism Score*	Quartile 1 (n = 129)	Quartile 2 (n = 128)	Quartile 3 (n = 128)	Quartile 4 (n = 129)
**Intrinsic Epigenetic Age Acceleration**	Mean Difference ß (95% CI)		Mean Difference ß (95% CI)	Mean Difference ß (95% CI)	Mean Difference ß (95% CI)
Unadjusted Model,	-0.02 (-0.47, 0.43)	Ref.	0.10 (-1.13, 1.33)	0.14 (-1.05, 1.33)	0.41 (-0.87, 1.69)
Confounders Model**	-0.02 (-0.51, 0.48)	Ref.	0.12 (-1.16, 1.40)	0.12 (-1.13, 1.36)	0.45 (-0.98, 1.89)
All Covariates Model***	-0.06 (-0.56, 0.44)	Ref.	0.09 (-1.22, 1.39)	0.12 (-1.15, 1.38)	0.54 (-0.92, 2.00)
**Extrinsic Epigenetic Age Acceleration**					
Unadjusted Model	-0.27 (-0.81, 0.28)	Ref.	0.73 (-0.75, 2.22)	0.56 (-0.88, 2.01)	0.98 (-0.57, 2.53)
Confounders Model**	-0.21 (-0.82, 0.41)	Ref.	0.60 (-0.99, 2.19)	0.53 (-1.02, 2.08)	0.58 (-1.21, 2.37)
All Covariates Model***	-0.17 (-0.80, 0.46)	Ref.	0.65 (-0.98, 2.29)	0.45 (-1.13, 2.03)	0.48 (-1.35, 2.31)

**Notes -** *Per 1 SD increase in LOT-R score

**Confounders model adjusts for: race, education, income, marital status, chronic conditions, depression,

***All covariates model additionally adjusts for: physical activity, smoking, BMI, diet, alcohol consumption, which could be potential confounders or intermediates

We also evaluated quartiles of optimism in relation to both EEAA and IEAA, in the WHI and NAS. Findings were null in both cohorts for IEAA. In the WHI women, in the unadjusted model, individuals in the lowest (versus highest) quartile of optimism had a lower mean EEAA score (β=-0.73; 95% CI: -1.32,-0.15; Table 3). However, this association was no longer statistically significant after adjusting for additional covariates, and all findings with EEAA were null in the NAS.

### Additional analyses

In both the WHI and NAS, after excluding individuals with depression, associations for optimism with either IEAA and EEAA remained consistently null (Appendix A 1, Table S3 and Table S4). Further, no statistically significant associations with IEAA and EEAA were evident for either the pessimism or the optimism subscales. Associations between the explanatory style optimism and both IEAA and EEAA were also uniformly null in all models (Table 4). In analyses stratified by race in WHI (Black and White women), associations remained consistently null across strata (data not shown). Findings from analyses that included the 736 WHI women who did not have the full set of covariates, were similarly null (data not shown). Finally, in WHI, after additionally adjusting for case-control status, WHI observational study membership, as well as clinical trial membership findings were uniformly null across all models (data not shown).

**Table 4:** Mean differences (Regression Coefficients) for association between optimism (PSM-R) and DNA methylation age in NAS (n=514).

	Optimism					
Outcome	Continuous Optimism Score*		Quartile 1 (n = 129)	Quartile 2 (n = 128)	Quartile 3 (n = 128)	Quartile 4 (n = 129)
**Intrinsic Epigenetic Age Acceleration**	Mean Difference ß (95% CI)			Mean Difference ß (95% CI)	Mean Difference ß (95% CI)	Mean Difference ß (95% CI)
Unadjusted Model	0.31 (-0.14, 0.76)	Ref.		0.46 (-0.80, 1.71)	0.13 (-1.12, 1.38)	1.16 (-0.11, 2.42)
Confounders Model**	0.32 (-0.18, 0.81)	Ref.		0.42 (-0.93, 1.78)	0.03 (-1.33, 1.38)	1.14 (-0.24, 2.52)
All Covariates Model***	0.29 (-0.21, 0.79)	Ref.		0.17 (-1.23, 1.57)	-0.04 (-1.42, 1.33)	1.06 (-0.36, 2.48)
**Extrinsic Epigenetic Age Acceleration**						
Unadjusted Model	-0.06 (-0.60, 0.49)	Ref.		-0.16 (-1.69, 1.37)	-1.04 (-2.56, 0.48)	-0.25 (-1.79, 1.28)
Confounders Model**	-0.11 (-0.72, 0.51)	Ref.		-0.20 (-1.89, 1.48)	-1.22 (-2.91, 0.47)	-0.47 (-2.19, 1.25)
All Covariates***	-0.04 (-0.67, 0.59)	Ref.		0.12 (-1.64, 1.87)	-0.92 (-2.64, 0.81)	-0.17 (-1.95, 1.60)

**Notes -** *Per 1 SD increase in Malinchoc optimism score (a higher score on this assessment indicates higher levels of pessimism, while a lower score indicates higher levels of optimism)

**Confounders model adjusts for: race, education, income, marital status, chronic conditions, depression,

***All covariates model additionally adjusts for: physical activity, smoking, BMI, diet, alcohol consumption, which could be potential confounders or intermediate

## DISCUSSION

We examined the association between optimism and epigenetic age acceleration measured by DNA methylation in two well characterized cohorts. We were able to examine two forms of optimism, dispositional and explanatory style. Regardless of which measure we considered and across cohorts, we found consistently null associations between optimism and both measures of DNA methylation aging, the intrinsic and extrinsic epigenetic age acceleration measure. While we did find one statistically significant association between the highest versus lowest quartile of dispositional optimism and lower EEAA score in an unadjusted model, it was evident only among the women. Thus, we remain cautious about interpreting this finding as occurring for reasons other than chance. Findings were unchanged in secondary analyses where we excluded those with depression, separately examined the optimism and pessimism subscales of the LOT, or considered another validated measure of optimism (PSM-R) available in the men.

There may be several explanations for these null associations. Our specific measures of epigenetic age acceleration may not be as relevant for optimism as other formulations might be. The DNA methylation age measures considered here reflect underlying aging processes that are at least partially under genetic control, and are more weakly associated with several lifestyle factors [27]. Pathway analysis suggests the components of the DNA methylation age [16,18] are enriched for immune cell trafficking and development, and these processes may not be strongly influenced by optimism. Further work is needed to evaluate other potential biologic mechanisms of optimism, both epigenetic and others, that may underlie the association of optimism with health. For example, one recently developed metric, DNA methylation PhenoAge, has a stronger association with lifestyle and wellness factors than the IEAA or EEAA measures [29], and as a result, may be more strongly linked to optimism. It is possible that other age acceleration scores that capture processes more tightly linked to optimism will also be developed. Further, although we did not observe evidence of a direct effect between optimism and DNAm age, future research should evaluate if optimism might moderate (or “dampen”) the effects of various stressors on DNAm age.

With increasing availability of epigenetic information, we will have additional opportunities to assess if the biologic correlates of optimism will be better captured by additional epigenetic metrics of aging [29,30], or if specific scores are less effective than broader agnostic analyses of the epigenome. Future studies that have repeated measures of DNA methylation aging could also provide a stronger test of the hypothesis that optimism influences epigenetic aging by evaluating if optimism is associated with changes in the rate of DNA methylation aging over time. However, another plausible explanation for our null findings could be that the biological pathways by which optimism works to reduce risk of age-related chronic diseases simply do not include changes in DNAm aging. Assessing these possibilities and alternative biological pathways for the observed association between optimism and chronic diseases of aging will be important next steps for this research.

The current study has some limitations. Both optimism and DNA methylation were assessed at a single point in time. Measurement error can be particularly problematic in studies such as ours, that rely on a single biomarker measurement [31], which likely has some random variability. Associations were cross-sectional and given the composition of each sample, findings may not be broadly generalizable, particularly to younger individuals. Nonetheless, this study has important strengths. We used two large and richly characterized cohorts and were able to assess associations in both men and women as well as adjust for potential confounders. Further, two forms of optimism, dispositional and explanatory style were assessed, with a commonly used validated measure dispositional optimism in both cohorts and a validated measure of explanatory style optimism in the NAS. Findings were remarkably similar across measures and cohorts. In conclusion, we examined associations between optimism and epigenetic aging in older men and women from two long-running cohorts with large sample sizes and found no statistically significant associations between optimism and intrinsic or extrinsic epigenetic age acceleration. Our findings may indicate that optimism is not specifically associated with biological mechanisms underlying these metrics of epigenetic age and age acceleration. It may also be that the combination of genes included in these epigenetic aging scores do not well-reflect the biological effects of optimism, and analyses of other clock scores, or broader agnostic or pathway analyses of DNA methylation could yield greater insight into biological processes underlying optimism.

Given robust and consistent findings that optimism is associated with reduced risk of developing a range of age-related diseases as well as overall mortality, and that the relationships are not fully explained by health behaviors [32], identifying novel pathways to improving health in aging remains an important goal. Our findings assess only one metric of epigenetic aging, but new methods and measures for assessing these processes are in active development (or recently available), suggesting continued effort to understand the range of epigenetic and other underlying biological mechanisms is warranted.

## MATERIALS AND METHODS

### Study Population

### *Women’s Health Initiative*

The Women’s Health Initiative (WHI) is a long-term study focused on identifying strategies for preventing major chronic diseases in postmenopausal women. Starting in 1993, racially and ethnically diverse women aged 50-79 years were recruited throughout the U.S. and entered either clinical trial(s) (WHI-CT; N=68,132) or the observational study (WHI-OS; N=93,676). Data were collected at 40 clinical centers throughout the country. At baseline, women completed self-administered questionnaires including information about sociodemographic factors, psychosocial characteristics, health behaviors, and chronic conditions. Further, after fasting overnight, they visited study clinics where certified clinical center staff collected blood specimens and performed anthropometric measurements.

The present study draws on data from two WHI substudies. The first substudy (Epigenetic Mechanisms of PM-Mediated Cardiovascular Disease Risk (EMPC; AS315; n=2,200) included a stratified random sample representative of the larger WHI CT population [27]. The second substudy (Integrative Genomics and Risk of Coronary Heart Disease and Related Phenotypes in the Women’s Health Initiative; BAA23; n=2,107) examined genomic determinants of coronary heart disease (CHD), using a nested case-control design (with oversampling of African Americans and Hispanics) in the WHI observational study and CT study populations. When data from both substudies were combined, there were 118 women who were in both substudies; thus, only data from EMPC was used when overlap existed (results were very similar when data from BAA23 were used instead). Further, 148 women were excluded because they had missing data on optimism and a further 7 were excluded due to missing data on the DNA methylation measures. Finally, some covariates were assessed only at baseline, and because some women had their blood drawn after baseline (n=736) they were excluded from primary analyses, resulting in a final analytic sample of 3,298 WHI women. In secondary analyses, we evaluated the main association of interest after including the 736 women, without fully control for covariates.

### *VA Normative Aging Study*

The VA Normative Aging Study (NAS) is a longitudinal investigation of normal aging processes in community-dwelling men, initiated in 1963. The study enrolled healthy men aged 21 to 81 years who were free of known chronic medical conditions. Men provided information on demographic factors, medical history, psychosocial factors, and lifestyle factors on a regular basis. They were interviewed at the VA Boston hospital every 3-5 years, and also participated in a physical exam and laboratory tests. Blood was drawn at each physical exam. Eligibility for this study required continued participation as of the time when DNA samples were first collected. Dropout has been < 1% per year in this cohort. Among the active 1,749 NAS participants at the time, DNA samples were collected from 1999-2009 for 774 participants. Among these, we excluded from analysis 260 men who did not have an optimism measurement before a DNA sample was taken. The final analytic sample included 514 NAS men with both DNA and measures of optimism.

Because restrictions apply to the public availability of these data, they are available from the WHI and NAS study coordinators upon reasonable request.

### Measures

### *Optimism assessment*

*WHI.* In WHI, dispositional optimism was assessed at baseline using the Life Orientation Test-Revised (LOT-R). The measure has demonstrated good discriminant and convergent validity, as well as good reliability [33]. Negatively worded items were reverse coded, and then all items were summed to create a composite score that ranged from 6 to 30, with higher scores indicating higher optimism. Following standard WHI protocol, the score was set to missing if study respondents were missing any of the LOT-R items. To facilitate comparisons of effect sizes across studies, we standardized optimism scores *(Mean(M)=0, Standard Deviation(SD)=1).* Internal consistency reliability was high in the analytic sample at baseline (Cronbach α=0.75). To assess the possibility of discontinuous or threshold effects, we also created quartiles of optimism based on the score distribution in the sample. Mean optimism scores by quartile were: 19, 23, 24, and 27. Following prior work on dispositional optimism [34], in secondary analyses, we also evaluated the optimistic (Cronbach α=0.77) and pessimistic (Cronbach α=0.74) subscales of LOT-R, each composed of three items from the overall scale.

*NAS.* In NAS, dispositional optimism was assessed using both the Revised Optimism-Pessimism Scale (PSM-R) and the original Life Orientation Test (LOT). The LOT is the parent scale from which the LOT-R was largely derived and includes 8 items that contribute to the scale score. Items were reverse coded as necessary and summed to create a composite score that ranged from 7 to 32 in this sample, with higher scores indicating higher optimism. Again, we standardized optimism scores *(M=0, SD=1).* Internal consistency reliability was high in the analytic sample at baseline (Cronbach α=0.78). We created quartiles of optimism based on the score distribution in the sample and mean optimism scores by quartile were: 16, 20, 22, and 26. In secondary analyses, we evaluated the optimistic (Cronbach α=0.75) and pessimistic (Cronbach α=0.80) subscales of LOT, each composed of four items from the overall scale.

Another measure of optimism based on explanatory style was assessed using the PSM–R, developed and validated by Malinchoc, Offord, and Colligan [35,36]. This bipolar scale measures the way individuals explain the causes of both good and bad events, and characterized explanatory style on a continuum from optimistic to pessimistic, by using 263 items selected from the revised Minnesota Multiphasic Personality Inventory (MMPI–2). Following the scoring algorithm, items were combined into a composite bipolar score reflecting a more optimistic explanatory style at the low end of the continuum and a more pessimistic one at the high end. This measure has predicted reduced risk of heart disease and slower lung function decline in the NAS cohort [3,37]. Internal consistency reliability was high in the analytic sample at baseline (Cronbach α=0.78), and prior research demonstrates this scale has high test-retest reliability of 0.90 [36]. To assess the possibility of discontinuous or threshold effects, we also created quartiles of optimism based on the score distribution in the sample. Mean scores by quartile were: 58, 47, 41, and 32.

### Assessment of DNA methylation and DNA methylation age acceleration

*WHI*. Methylation analysis for both substudies was performed using the Illumina Infinium Human-Methylation450 Beadchip, which measures single-CpG resolution DNA methylation levels at 485,577 unique CpG sites in the human genome. The BAA23 WHI substudy samples were processed at the HudsonAlpha Institute of Biotechnology. The EMPC WHI substudy samples were processed at the Northwestern University Genomics Core. All measurements were quality-controlled and batch adjusted as described elsewhere [27].

*NAS*. Methylation data were generated at the Northwestern University Genomics Core Facility. All measurements underwent quality-control. The Bioconductor minfi package Illumina-type background correction without normalization was used to preprocess the samples and generate methylation beta values to compute DNAm-age [38].

*Epigenetic age acceleration scores*. In all of the analyses, we considered both the intrinsic epigenetic age acceleration score (IEAA) [16] and the extrinsic epigenetic age acceleration score (EEAA) [15]. The two scores were calculated identically in WHI and NAS based on scripts developed by Horvath (the IEAA variable is denoted as *AAHOAdjCellCounts* and the EEAA variable is denoted as *BioAge4HAStaticAdjAge*). Both the EEAA and IEAA measures represent accelerated biological aging that exceeds chronological age, with positive values indicating that epigenetic age is higher than chronological age. Both measures are calculated by obtaining the residual when regressing DNAm age on chronological age. Intrinsic epigenetic age acceleration captures properties of aging that are independent of cell type and organ whereas extrinsic epigenetic acceleration likely reflects both epigenetic variation and age-related changes in cell distributions in blood [27]. In WHI, the correlation between IEAA and EEAA was 0.36 and in NAS the correlation was 0.32.

### Assessment of potential confounders and other covariates

*WHI*. Potential confounders included sociodemographic factors and depression. Sociodemographic variables were obtained from the baseline questionnaire and included age (continuous), race (White, Black/African-American, Hispanic/Latino, Other), marital status (married/marriage like-relationship, divorced/single, widowed), education (less than high school, high school graduate, some college or associate degree, college or more), income (<$20,000, $20,000-$49,999, $50,000-$74,999, $75,000+, don’t know). Depression status (yes/no) was defined according to Burnam Screening Algorithm questionnaire that includes 6 items from the Center for Epidemiologic Studies Depression Scale (CES-D) and two from the Diagnostic Interview Scale (DIS) [39,40], with a cutoff of ≥0.06 indicating depression [39]. Health conditions were self-reported (yes/no) and included: hypertension, high cholesterol, cardiovascular disease, diabetes, stroke, cancer. Height and weight were measured by trained staff and used to calculate Body Mass Index (BMI) in kg/m^2^. and then, three BMI categories were created (<24.9, 25.0 to 29.9, ≥30.0). Potential confounding or intermediate variables included the following health behaviors, all self-reported on the baseline questionnaire: cigarette smoking (never, former, current smoker), physical activity (weekly expenditure of metabolic equivalent tasks (METs; <3 METs/week, 3-<9 METs/week, 9-<19 METs/week, 18-<27 METs/week, 27+ METs/week), alcohol intake (non-drinker, current drinker), diet (122-item food frequency questionnaire (FFQ) [41]; overall diet quality was quantified using the Alternative Healthy Eating Index (scale 0-110; the AHEI includes 11 different diet components).

*NAS*. Potential confounders included sociodemographic factors, depression, and chronic conditions. Sociodemographic variables were obtained from the baseline questionnaire and included age (continuous), educational attainment (years), race/ethnicity (white/non-white), marital status (married/not married). Depressive symptoms were assessed with a subscale from the Brief Symptom Inventory [42], with a cutoff of ≥0.638 indicating depression. Information about health conditions was self-reported (yes/no), updated every 3-5 years, and included: coronary heart disease, stroke, diabetes, and cancer. BMI in kg/m^2^ was calculated from weight and height measured by study staff (<24.9, 25.0-29.9, ≥30.0). Further variables included the following health behaviors (updated every 3-5 years), which could be confounders or intermediates: cigarette smoking status (current, former, never), physical activity (created based on questions asking about energy expenditure (e.g., frequency of sports activities, flights of stairs climbed/day, distance walked, etc., to calculate the following metabolic equivalent of tasks (METs) categories; <3 METs/week, 3-<9 METs/week, 9-<19 METs/week, 18-<27 METs/week, 27+ METs/week), alcohol intake, (<2 drinks versus 2+ drinks), and diet (frequency of fruit and vegetable consumption).

### Statistical analysis

In primary analyses, we considered optimism as a continuous standardized variable, where associations represent the change in DNAm age as a function of a 1 standard deviation increase in optimism, and also evaluated optimism categorized into quartiles (based on the sample-specific distribution of scores). All models were run separately within each cohort. Several sets of models were tested using ordinary least square regression. The first model did not adjust for any covariates. The second model added basic potential confounders including race, education, income, marital status, chronic conditions, and depression. A third model further added variables that could be potential confounders or intermediates including physical activity, smoking, BMI, diet, and alcohol consumption.

We conducted several secondary analyses. First, to test potential residual confounding due to depression, we excluded those with high levels of depressive symptoms. Second, to evaluate whether associations differed by the optimism or pessimism subscale, we separately evaluated these two subscales. Third, to evaluate the consistency of results across different measures of optimism, we conducted analyses in NAS using the PSM-R instead of the LOT. Fourth, In WHI we conducted stratified analyses in Black and White women. Fifth, we evaluated findings after adding to our sample 736 WHI women who had blood draws after the baseline assessment, and who did not have full covariate data. Finally, to evaluate potential confounding caused by case-control status (CHD-no CHD over follow-up), WHI observational study membership, or clinical trial membership (hormone therapy (HT), dietary modification (DM), or calcium and Vitamin D supplementation (CaD)), we adjusted for all of these factors in the WHI cohort.

All analyses were completed using Stata (StataCorp. 2017. Stata Statistical Software: Release 15.0. College Station, TX: StataCorp LP) or R 3.4.1 (R Core Team (2017). R Foundation for Statistical Computing, Vienna, Austria).

### Ethics Approval

For WHI, institutional review board approval was obtained at each clinical center and all participants provided written informed consent. For NAS, participants provided written informed consent to the VA Institutional Review Board.

### Availability of data and materials

The data that support the findings of this study are available upon reasonable request to Ron Spiro (aspiro3@bu.edu) for NAS data. Details regarding access to all WHI data are available at: https://www.whi.org/researchers/data/Pages/Home.aspx."

## Supplementary Material

Supplementary Tables
