# Supplementary material for: Optimism is not associated with two indicators of DNA methylation aging
Source: Aging (Albany NY). 2019 Jul 18;11(14):4970–89. doi: 10.18632/aging.102090 (PMC6682522; doi:10.18632/aging.102090)
Supplement: Supplementary Tables [file aging-11-102090-s001.pdf]

SUPPLEMENTARY MATERIAL

Table S1. Characteristics of Women's Health Initiative participants at baseline (n=3,298).

	**Optimism Score**			
**Characteristic**	Quartile 1 (n = 1,004)	Quartile 2 (n = 809)	Quartile 3 (n = 732)	Quartile 4 (n = 753)
**Demographic Factors**				
Mean Age (SD)	63.4 (7.2)	63.8 (7.0)	64.0 (7.0)	63.2 (7.2)
Race/Ethnicity (%)				
White	43.7	51.6	57.1	51.9
Black / African-American	28.1	28.4	27.2	33.2
Hispanic / Latino	23.7	16.7	13.0	12.4
Other	4.5	3.3	2.7	2.5
Missing	0	0	0	0
Marital Status (%)				
Marriage or marriage-like relationship	50.6	59.8	56.8	57.2
Divorced or single	25.0	20.0	20.9	23.5
Widowed	23.4	19.7	21.7	19.0
Missing	1.0	0.5	0.6	0.3
Education (%)				
Less than high school	32.4	24.2	23.4	14.6
High school graduate	22.2	20.0	17.5	13.9
Some college or associate degree	24.0	24.7	26.1	28.7
College or more	20.5	30.2	32.5	41.8
Missing	0.9	0.9	0.6	0.9
Income (%)				
Less than $20,000	35.2	25.0	22.1	17.4
$20,000 to $49,999	41.7	45.2	43.7	44.5
$50,000 to $74,999	9.1	14.8	20.2	16.2
$75,000 or more	6.6	9.2	10.5	15.7
Missing	7.5	5.8	3.4	6.2
**Health Factors**				
Depressed (%)*				
Not depressed	73.5	90.2	90.6	93.4
Depressed	20.3	7.2	7.0	4.0
Missing	6.2	2.6	2.5	2.7

Chronic Condition (%**)				
No chronic condition	36.7	36.7	37.2	39.8
Chronic condition	52.1	52.0	49.5	49.4
Missing	11.3	11.3	13.4	10.8
**Health Behaviors**				
Smoking (%)				
Never smoker	52.5	53.2	49.5	52.6
Past smoker	34.7	36.6	41.4	37.3
Current smoker	12.1	8.2	7.9	8.6
Missing	0.8	2.1	1.2	1.5
Physical activity level (METS/week; %)				
<3.0	41.6	32.6	32.8	32.3
3.0-8.99	23.5	24.0	21.5	22.7
9.0-17.99	14.4	19.0	18.9	19.5
18.0-26.99	7.1	7.5	8.9	9.4
≥27	6.4	9.9	8.7	9.7
Missing	7.0	6.9	9.3	6.4
Mean Diet (Healthy Eating Index; SD)	63.6 (11.5)	65.7 (11.3)	64.7 (11.7)	65.7 (11.2)
Current drinker (%)				
Non drinker	42.2	36.2	35.0	36.3
Current drinker	57.1	62.8	64.1	63.4
Missing	0.7	1.0	1.0	0.4
Body Mass Index (%)				
Normal (<24.9)	19.6	22.3	25.7	24.0
Overweight (25.0-29.9)	35.6	35.5	33.5	32.3
Obese (≥30.0)	44.3	41.5	40.6	42.6
Missing	0.5	0.7	0.3	1.1

Notes- *Depressive symptoms were measured using the Burnam Screening Algorithm, a questionnaire that includes 6 items from the Center for Epidemiologic Studies Depression Scale (CES-D) and two from the Diagnostic Interview Scale (DIS), with a cutoff of ≥0.06 indicating depression

**Chronic conditions include: 1) hypertension, 2) high cholesterol, 3) cardiovascular disease, 4) diabetes, 5) stroke, 6) cancer

Table S2. Characteristics of Normative Age Study (NAS) participants at baseline (n=514).

	Optimism Score			
**Characteristic**	Quartile 1 (n = 129)	Quartile 2 (n = 128)	Quartile 3 (n = 128)	Quartile 4 (n = 129)
**Demographic Factors**				
Mean Age (SD)	72.9 (6.6)	72.3 (6.7)	72.8 (6.7)	73.2 (6.4)
Race/Ethnicity (%)				
White	99.2	100	99.2	99.2
Black / African-American	0.8	0	0	0.8
Hispanic / Latino	0	0	0.8	0
Other	0	0	0	0
Missing	0	0	0	0
Marital Status (%)				
Marriage or marriage-like relationship	73.6	75.8	78.9	74.4
Divorced or single	16.3	12.5	11.7	15.5
Widowed	9.3	10.2	8.6	10.1
Missing	0.8	1.6	0.8	0
Education (%)				
Less than high school	5.4	6.2	1.6	2.3
High school graduate	20.9	19.5	20.3	20.9
Some college or associate degree	24.8	13.3	16.4	16.3
College or more	27.9	34.4	37.5	39.5
Missing	20.9	26.6	24.2	20.9
Income (%)				
Less than $60,000	29.5	30.5	23.4	26.4
$60,000 to $69,999	19.4	20.3	19.5	19.4
$70,000 to $89,999	21.7	21.9	14.1	25.6
$90,000 or more	25.6	26.6	43.0	27.9
Don't know	0	0	0	0
Missing	3.9	0.8	0	0.8
**Health Factors**				
Depressed (%)*				
Not depressed	68.2	89.8	84.4	96.1
Depressed	28.7	7.0	10.9	0.8
Missing	3.1	3.1	4.7	3.1
Chronic Condition (%**)				
No chronic condition	48.1	59.4	59.4	64.3
Chronic condition	51.9	40.6	40.6	35.7
Missing	0.0	0.0	0.0	0.0

**Health Behaviors**				
Smoking (%)				
Never smoker	28.7	35.9	25.0	33.3
Past smoker	69.0	58.6	72.7	62.8
Current smoker	2.3	5.5	2.3	3.9
Missing	0	0	0	0
Physical activity level (METS/week; %)				
<3.0	38	19.5	30.5	19.4
3.0-8.99	27.1	36.7	32.0	29.5
9.0-17.99	20.2	12.5	14.1	19.4
18.0-26.99	4.7	10.9	10.2	10.1
≥27	10.1	19.5	13.3	21.7
Missing	0	0.8	0	0
Mean Fruit Intake (SD)	2.6 (1.8)	2.4 (1.5)	2.8 (1.7)	2.5 (1.6)
Mean Vegetable Intake (SD)	3.1 (1.9)	3.3 (2.2)	3.6 (2.4)	3.5 (2)
Current drinker (%)				
Non drinker	29.5	27.3	22.7	19.4
Current drinker	69.8	68.8	75.0	79.8
Missing	0.8	3.9	2.3	0.8
Body Mass Index (%)				
Normal (<24.9)	16.3	20.3	20.3	21.7
Overweight (25.0-29.9)	50.4	55.5	50.0	56.6
Obese (≥30.0)	33.3	24.2	29.7	21.7
Missing	0	0	0	0

Notes- *Depressive symptoms were measured using the Brief Symptom Inventory (BSI), with a cutoff of ≥0.638 indicating depression
**Chronic conditions include: 1) cardiovascular disease, 2) diabetes, 3) stroke, 4) cancer

Table S3. Mean differences (Regression Coefficients) for association between optimism and DNA methylation age in Women's Health Inititative, after excluding women with depression (n=2,834)*

	Optimism				
	Continuous	Quartile 1	Quartile 2	Quartile 3	Quartile 4
Outcome	Optimism Score[b]	(n = 738)	(n = 730)	(n = 663)	(n = 703)
**Horvath Clock Score (IEAA)**					
Confounders Model[c]	-0.02 (-0.21, 0.18)	Ref.	-0.24 (-0.75, 0.27)	-0.21 (-0.73, 0.32)	-0.05 (-0.57, 0.48)
**Hannum Clock Score (EEAA)**					
Confounders Model[c]	0.00 (-0.25, 0.24)	Ref.	0-.10 (-0.73, 0.53)	-0.43 (-1.09, 0.22)	-0.06 (-0.71, 0.59)

Notes- *All models adjusted for WHI substudy (EMPC or BAA23)
**Per 1 SD increase in LOT-R score

Table S4. Mean differences (Regression Coefficients) for association between optimism and DNA methylation age in Normative Age Study (NAS), after excluding men with depression (n=435).

	Optimism				
	Continuous	Quartile 1	Quartile 2	Quartile 3	Quartile 4
Outcome	Optimism Score[a]	(n = 88)	(n = 115)	(n = 108)	(n = 124)
**Horvath Clock Score (IEAA)**					
Confounders Model[b]	-0.06 (-0.61, 0.48)	Ref.	0.18 (-1.18, 1.55)	0.31 (-1.03, 1.66)	0.37 (-1.26, 2.00)
**Hannum Clock Score (EEAA)**					
Confounders Model[b]	-0.26 (-0.94, 0.43)	Ref.	0.28 (-1.64, 2.20)	-1.12 (-2.98, 0.74)	0.52 (-1.32, 2.36)

Notes- *Per 1 SD increase in LOT score
**Confounders model adjusts for: race, education, income, marital status, chronic conditions, depression
